# Overexpression of Prolyl-4-Hydroxylase-*α*1 Stabilizes but Increases Shear Stress-Induced Atherosclerotic Plaque in Apolipoprotein E-Deficient Mice

**DOI:** 10.1155/2016/1701637

**Published:** 2016-10-12

**Authors:** Xiao-qing Cao, Xin-xin Liu, Meng-meng Li, Yu Zhang, Liang Chen, Lin Wang, Ming-xue Di, Mei Zhang

**Affiliations:** Key Laboratory of Cardiovascular Remodeling and Function Research, Chinese Ministry of Education and Chinese Ministry of Public Health, Department of Cardiology, Qilu Hospital of Shandong University, Ji'nan 250012, China

## Abstract

The rupture and erosion of atherosclerotic plaque can induce coronary thrombosis. Prolyl-4-hydroxylase (P4H) plays a central role in the synthesis of all known types of collagens, which are the most abundant constituent of the extracellular matrix in atherosclerotic plaque. The pathogenesis of atherosclerosis is thought to be in part caused by shear stress. In this study, we aimed to investigate a relationship between P4H*α*1 and shear stress-induced atherosclerotic plaque. Carotid arteries of ApoE−/− mice were exposed to low and oscillatory shear stress conditions by the placement of a shear stress cast for 2 weeks; we divided 60 male ApoE−/− mice into three groups for treatments with saline (mock) (*n* = 20), empty lentivirus (lenti-EGFP) (*n* = 20), and lentivirus-P4H*α*1 (lenti-P4H*α*1) (*n* = 20). Our results reveal that after 2 weeks of lenti-P4H*α*1 treatment both low and oscillatory shear stress-induced plaques increased collagen and the thickness of fibrous cap and decreased macrophage accumulation but no change in lipid accumulation. We also observed that overexpression of P4Ha1 increased plaque size. Our study suggests that P4H*α*1 overexpression might be a potential therapeutic target in stabilizing vulnerable plaques.

## 1. Introduction

Coronary thrombosis, induced by plaque rupture and plaque erosion, can cause acute coronary syndromes (ACS) such as unstable angina, acute myocardial infarction, and sudden coronary death [1]. Histopathologically, the plaque vulnerability is enhanced if plaque contains a large lipid-rich necrotic core covered by a thin fibrous cap [2–4]. As the main components of plaque's fibrous cap, collagen determines the plaque vulnerability [5, 6]. Furthermore, the physical state of collagen can have a great impact on the development of atherosclerotic plaque by interfering with SMCs and macrophages [7, 8].

Prolyl-4-hydroxylase (P4H) plays a central role in the synthesis of all known types of collagens [9]. As an isoenzyme of P4H, P4H*α*1 is a rate-limiting enzyme and is essential for collagen maturation and secretion [10]. It has been shown that manipulating expression of P4H*α*1 affects atherosclerotic plaque stabilization, while different manipulations of P4H*α*1 have different influences on lesional collagen synthesis [11–13].

Shear stress, the force of blood flow on the endothelium, is thought to play a critical role in the pathogenesis of cardiovascular diseases, such as atherosclerosis. Cheng et al. [14] developed a perivascular shear stress modifier (called a cast) which could induce changes in shear stress patterns in a straight vessel for studying the development of carotid atherosclerotic lesions of stable and vulnerable phenotypes in apolipoprotein E-deficient (ApoE−/−) mice. Using this technique, stable lesions develop distal to the cast under conditions of oscillatory shear stress, whereas vulnerable lesions (containing a small amount of collagen) develop proximal to the cast due to a low shear stress.

In the current study, we tested the hypothesis that P4Ha1, a key factor for regulating collagen, affects the stabilization of shear stress-induced atherosclerotic plaque. Our study data suggested a new therapeutic target candidate to attenuate coronary thrombosis.

## 2. Materials and Methods

### 2.1. Animals

Male ApoE−/− mice (*n* = 60), 8 weeks old, were obtained from Beijing University Animal Research Center. They were housed in standard cages on 12 h light/12 h dark cycle with free access to water and food. All experimental procedures were in compliance with the* Guide for the Care and Use of Laboratory Animals*, published by the US National Institutes of Health (NIH Publication number 85-23, revised 1985) and Shandong University.

### 2.2. Shear Stress Modifier Placement and Grouping

All 60 mice were given a high-fat diet containing 0.25% cholesterol and 15% cocoa butter, starting at 2 weeks before surgery. As described previously by Cheng et al. [14], we used a shear stress cast that imposed a fixed geometry on the right common carotid artery to induce standardized changes in shear stress, which resulted in a gradual stenosis. Sixty mice were randomly assigned to three groups (*n* = 20 in each group): mock group; lenti-EGFP group; and lenti-P4Ha1 group. Two weeks after cast placement, the mice in each group were given a tail vein injection of saline, empty lentivirus (3.5 × 10^6^ TU null lentivirus), or lentivirus-P4H*α*1 (3.5 × 10^6^ TU lentivirus containing P4H*α*1) transgenic therapy, respectively. The mice were killed at 4 weeks after cast placement. Blood samples were taken before and after injection to detect the levels of total cholesterol, low-density lipoprotein (LDL) cholesterol, and hydroxyproline.

### 2.3. Immunohistochemistry and Histology

To compare the effect of low shear stress on lesion formation in the three groups, the mice were humanely euthanized at 4 weeks after surgery, followed by transcardial perfusion with phosphate-buffered saline and 4% paraformaldehyde. The right common carotid arteries were carefully stripped and fixed in 4% paraformaldehyde overnight. The tissues were frozen in OCT compound and then cut at 5 *μ*m thickness continuously. The serial cryosections were stained with hematoxylin (Sigma, St. Louis, MO, USA) and eosin (Merck, Whitehouse Station, NJ, USA). The sirius red staining was used for collagen and Oil Red 0 (Sigma) staining was used for lipid-rich lesions. The macrophages were detected with anti-rabbit CD68 (dilution 1 : 100, Proteintech, Chicago, USA) by immunohistochemistry.

### 2.4. RNA Extraction and Quantitative Real-Time PCR

The carotid artery specimens after low and oscillatory shear stress were pooled for RNA isolation with TriZol (Invitrogen) according to the manufacturer's instructions. The concentration of total RNA was tested by spectrophotometry and reverse-transcribed into cDNA. The PCR was performed to determine the gene expression using SYBR Green Technology (Bio-Rad, USA), and the housekeeping gene was used as an internal control. The sequences of primers for *β*-actin were as follows: forward: 5′-TGGAACGGTGAAGGTGACA-3′, reverse: 5′-GGACTTCCTGTAACAATGCA-3′; the sequences of primers for P4Ha1 were as follows: forward: 5′-ATGACCCTGAGACTGGAAA-3′, reverse: 5′-GCCAGGCACTCTTAGATACT-3′. The data were estimated by the 2^−ΔΔCT^ method. All experiments were repeated for three times and only the mean value was used for analysis.

### 2.5. Western Blot Analysis

The right carotid arteries, after low and oscillatory shear stress treatment, were collected for protein extraction. The equal amounts of protein were separated with a 10% SDS-PAGE and transferred to polyvinylidene difluoride membranes (Millipore, Plano, TX, USA). The membrane was blocked with 5% nonfat milk in TBS-T (50 mmol/Tris, pH 7.5, 150 mmol/L NaCl, 0.05% Tween-20) for 2 h at room temperature and then washed in TBS-T 3x for 10 min. After wash, the blots were incubated overnight at 4°C with an appropriate primary antibody. The membranes were then washed with TBS-T and incubated with horseradish peroxidase-conjugated secondary antibodies for 2 h at room temperature. After 3 washes in TBS-T, the membranes were visualized with ECL plus reagents (Millipore, Plano, TX, USA).

The primary antibodies used in the experiments were goat anti-P4H*α*1 (1 : 500, Abcam, Cambridge, UK), rabbit anti-TGF-*β*1 (1 : 500, Proteintech, Chicago, USA), and rabbit anti-*β*-actin (1 : 1000, Cell Signaling Technology, Boston, MA, USA).

### 2.6. Statistical Analysis

All data are expressed as mean ± SD. SPSS for Windows, and version 16.0 (SPSS, Chicago, IL, USA) was used for statistical analysis. When comparing more than two groups, one-way ANOVA was used and the least significant difference test to further compare the two groups for significant differences. A group difference was considered significant when *p* < 0.05.

## 3. Results

### 3.1. TC, TG, LDL-C, HDL-C, and Hydroxyproline Concentrations

During the experiments, one mouse in the mock group and two in the lenti-P4Ha1 group died. Thus, the sample sizes were 19, 20, and 18 for the three groups, respectively. The body weight and level of serum lipids did not differ between virally transfected and sham mice (*p* > 0.05, Table 1). However, the level of hydroxyproline in lenti-P4H*α*1 group was much higher than the other two groups (*p* < 0.05, Table 1). Therefore, empty lentivirus or lentivirus-P4Ha1 had no significant effect on lipid levels in the circulation system, suggesting that diet or lipid levels did not appear to play a role in the atherosclerotic lesion differences between the three groups.

### 3.2. Overexpression of P4Ha1 Increased Plaque Size

**Figure 1:**
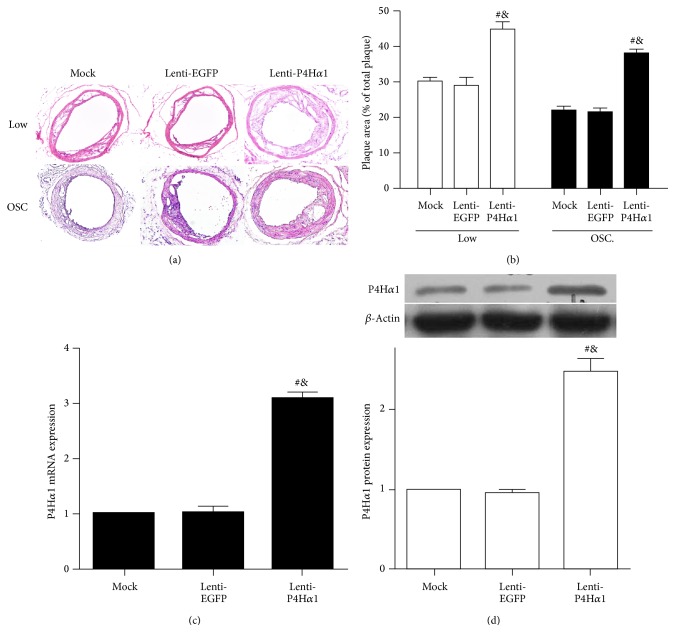
Effect of P4Ha1 overexpression on plaque size and the efficiency of lentivirus-P4Ha1 in mice. (a) Cross sections of carotid arteries stained with hematoxylin and eosin. (b) Analyses of plaque area. (c) RT-PCR analysis of P4Ha1 mRNA expression with lenti-P4Ha1, lenti-EGFP, and mock treatment. (d) Western blot analysis of P4Ha1 protein expression with lenti-P4Ha1, lenti-EGFP, and mock treatment (^#^ *p* < 0.01 versus mock; ^&^ *p* < 0.01 versus lenti-EGFP).

To test our hypothesis that altered collagen contents by P4Ha1 overexpression affects the development of atherosclerotic plaque, we studied the development of carotid atherosclerotic lesions of vulnerable and stable phenotypes induced by a perivascular cast [14]. Using this technique, stable lesions develop under oscillatory shear stress, whereas vulnerable lesions develop under low shear stress. The lesion size under low and oscillatory shear stress was increased in lenti-P4H*α*1 mice (*p* < 0.01, Figures 1(a) and 1(b)).

To determine the transfection efficiency of lentivirus-P4Ha1 in mice, we measured the expressions of P4Ha1 in different regions using RT-PCR and western blot. The mRNA and protein expressions of P4H*α*1 were higher in lenti-P4Ha1 mice than in mock or lenti-EGFP mice (*p* < 0.01, Figures 1(c) and 1(d)).

### 3.3. Collagen Content Was Increased in P4Ha1-Overexpressed Plaques

**Figure 2:**
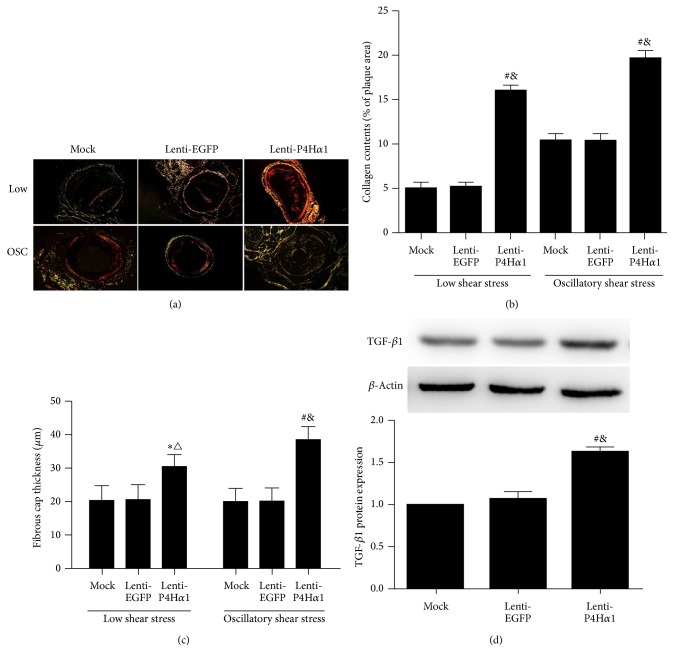
Effect of P4Ha1 overexpression on collagen content in plaques. (a) Representative collagen staining in the two different shear stress regions in three groups of mice. (b) Quantitative analysis of plaque collagen contents in the two different shear stress regions in three groups of mice. (c) Quantitative analysis of fibrous cap thickness in the two different shear stress regions in three groups of mice. (d) Western blot analysis of TGF-*β*1 protein expression with lenti-P4Ha1, lenti-EGFP, and mock (^*∗*^ *p* < 0.05 versus mock; ^#^ *p* < 0.01 versus mock; ^△^ *p* < 0.01 versus lenti-EGFP; ^&^ *p* < 0.01 versus lenti-EGFP).

The role of collagens in atherogenesis is double-edged. That is, defective fiber assembly or degradation can cause plaque rupture and subsequent thrombosis, whereas an excessive collagen production can promote plaque growth and thereby contributes to vascular stenosis [15]. We measured the thickness of fibrous cap and collagen content in shear stress-induced carotid plaques. Sirius red staining showed that P4H*α*1 overexpression increased the thickness of fibrous cap and collagen content in both low and oscillatory shear stress-induced carotid plaques as compared to controls (*p* < 0.01, *p* < 0.05, Figures 2(a), 2(b), and 2(c)). Furthermore, we failed to find any difference between two control groups, that is, mock and lenti-EGFP mice.

Transforming growth factor beta-1 (TGF-*β*1) is known to be a major profibrotic cytokine that plays a critical role in matrix remodeling and collagen synthesis [16]. To determine whether TGF-*β*1 plays a role in atherosclerotic plaque formation in conjunction with lenti-P4Ha1 treatment, we measured the expression levels of TGF-*β*1 in carotid plaques using western blot. The protein expressions of TGF-*β*1 were higher in lenti-P4Ha1 mice than in mock or lenti-EGFP mice (*p* < 0.01, Figure 2(d)).

### 3.4. No Difference of Lipid Accumulation in P4Ha1-Overexpressed Plaques

**Figure 3:**
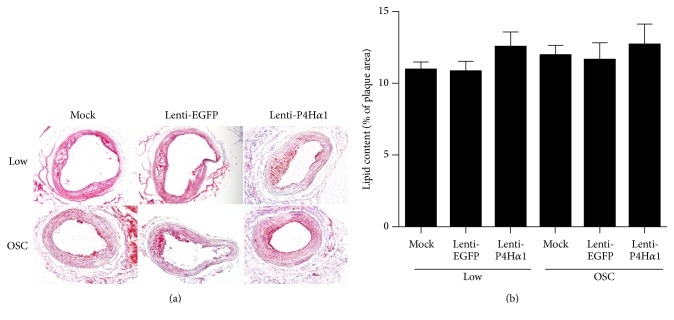
Effect of P4Ha1 overexpression on lipid accumulation in plaques. (a) Representative Oil Red O staining in different shear stress regions in three groups of mice. (b) Quantitative analysis of plaque lipid accumulation in different shear stress regions in three groups of mice.

Lipid retention is another essential process in atherosclerosis development [17], and Type I collagen can influence LDL-retention [18]. Oil Red 0 staining was used to measure the lipid content in the shear stress-induced plaques. However, the lipid accumulation did not differ among all three groups (*p* > 0.05, Figures 3(a) and 3(b)).

### 3.5. Overexpression of P4Ha1 Reduced Macrophage Infiltration

**Figure 4:**
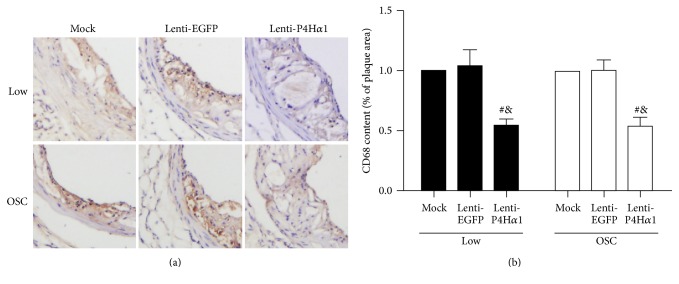
Effect of P4Ha1 overexpression on macrophage accumulation in plaques. (a) Representative immunohistochemical staining (CD68) in different shear stress regions in three groups of mice. (b) Quantitative analysis of CD68 content in different shear stress regions in three groups of mice (^#^ *p* < 0.01 versus mock; ^&^ *p* < 0.01 versus lenti-EGFP).

As macrophages play an important role in the progression of atherosclerotic plaque [19, 20] and can promote formation of unstable plaques by maintaining proinflammatory microenvironment [21], we investigated the infiltration of macrophages in plaque tissues by being stained with macrophage marker CD68. Compared to controls, overexpression of P4H*α*1 significantly reduced the expression of CD68 in both low and oscillatory shear stress-induced carotid plaques (*p* < 0.01, Figures 4(a) and 4(b)). However, there were no significant differences in expression of CD68 between two control groups, that is, mock and lenti-EGFP mice.

## 4. Discussion

Shear stress, particularly the low and oscillatory shear stress, is widely assumed to play a crucial pathophysiological role in coronary atherosclerosis. Recent studies [14] have revealed that patterns of shear stress can affect the size and phenotype of the lesions. In animal models, atherosclerotic plaques induced by low shear stress appear to be a vulnerable phenotype. As a rupture of the vulnerable atherosclerotic plaques is the major cause of mortality in humans, our animal results may have a strong implication on the pathogenesis of coronary atherosclerosis. In particular, we for the first time demonstrated that P4H*α*1 overexpression affected the size and composition of shear stress-induced plaques.

Collagens are the most abundant constituent of the extracellular matrix in atherosclerotic plaques and play a crucial role in keeping the atherosclerotic plaques intact and stable. P4H*α*1, which is essential for collagen biogenesis, plays a central role in the synthesis of all known types of collagens [9, 10]. P4H*α*1 catalyzes the formation of hydroxyproline that is required for folding newly synthesized procollagen polypeptide chains into stable triple helical molecules [22, 23]. Our previous works [11, 12] have shown that P4H*α*1 expression decreases gradually during the pathological course of atherosclerotic plaques and the decreased expression of P4H*α*1 correlates with atherosclerotic plaque instability. In the present study, we attempted to elucidate a relationship between an enhanced level of lesional P4H*α*1 and shear stress-induced atherosclerotic plaques in ApoE−/− mouse. We used a slow viral vector that successfully delivered P4H*α*1 gene into the atherosclerotic plaque as demonstrated by enhanced mRNA and protein expressions of P4H*α*1 and an increase in plasma level of hydroxyproline in lenti-P4H*α*1 mice.

Previous studies [24] have suggested that plaque stability depends upon the plaque composition and state of the fibrous cap. The tensile strength of the intima increases as a rupture of the fibrous cap occurs. The ability of the atherosclerotic plaques to resist mechanical tensile force increases as the collagen component in plaques increases, whereas an increase in the lipid component reduces the plaques' resistance to mechanical tensile force. Therefore, the ratio of lipid to collagen content is a useful index for evaluating plaque stability [25]. Here, we found that overexpression of P4H*α*1 increased collagen content in both low and oscillatory shear stress regions. Interestingly, we failed to find any change in lipid accumulation. As such, overexpression of P4H*α*1 appeared to decrease the ratio of lipid to collagen in both low and oscillatory shear stress-induced plaques. In addition, macrophages play a pivotal role in atherosclerotic plaque destabilization [26, 27]. Increased macrophages infiltration is a hallmark of atherosclerotic plaques prone to acute complications [28]. Our data suggested that overexpression of P4H*α*1 could significantly decrease the accumulation of macrophages in both low and oscillatory shear stress-induced plaques.

The lesional fibrous cap is an important factor of stability of atherosclerotic plaques. The lesional fibrous cap mainly consists of Types I and III collagens. We showed that lentivirus-mediated overexpression of P4H*α*1 increased the collagen content and thickened the fibrous cap in low and oscillatory shear stress-induced plaques.

In addition, we found that overexpression of P4H*α*1 increased the size of the atherosclerotic plaques formed in both low and oscillatory shear stress regions. To the best of our knowledge, this was the first evidence that P4H*α*1 overexpression regulates the process of atherogenesis under low/oscillatory shear stress. That is, overexpression of P4H*α*1 appeared to accelerate atheromatous plaque formation in the ApoE−/− mice. These findings are consistent with our previous results [11], where overexpression of P4H*α*1 accelerated aortic lesion formation in ApoE−/− mice. It is noteworthy that previous studies did not elucidate a relationship between P4H*α*1 and atherosclerotic plaques formation under shear stress.

Transforming growth factor beta-1 (TGF-*β*1) has an essential role in atherogenesis [29–31]. Some studies have shown that TGF-*β*1 could accelerate atherosclerosis by increasing extracellular matrix (ECM) accumulation [32, 33]. In the present study, we showed that overexpression of P4H*α*1 increased the level of TGF-*β*1 in carotid plaques. We speculate that elevated expression of TGF-*β*1 in atheroma lesions may be partly correlated with plaques growth in ApoE−/− mice with P4H*α*1 overexpression. Thus, overexpression of P4H*α*1 promotes fibrosis via increasing the collagen content and possibly ECM accumulation in shear stress-induced plaques. However, whether increasing atheroma fibrosis would be beneficial or detrimental still needs to be determined. On the one side, enhanced fibrosis would stabilize the plaque with a high extracellular matrix content, which may help to reduce plaque vulnerability and make plaque rupture less likely. On the other side, enhanced fibrosis augments plaque size, which leads to vascular stenosis and an increased likelihood of clinical symptoms of atherosclerosis. Therefore, increasing atheroma fibrosis shows a double-edged sword effect on atherogenesis.

From a therapeutic standpoint, our results have a clinical implication on treating shear stress-related atherosclerosis. Modulating the expression of P4H*α*1 appears a novel approach to increase the stability of atherosclerotic plaques. Our previous studies [12] showed that downregulation of P4H*α*1 by IL-6 destabilizes atherosclerotic plaques, while upregulation of P4H*α*1 by adiponectin increases collagen production that lowers the vulnerability of plaques [13]. Together with our current results, P4H*α*1 overexpression appeared a potential therapeutic target to stabilize vulnerable plaques. But future studies are needed to determine how to prevent vascular stenosis due to an excessive collagen production.

## 5. Conclusion

In summary, our study identified that overexpression of P4H*α*1 may increase the stability of shear stress-induced plaques in ApoE-deficient mice. This result provides supporting evidence that P4H*α*1 overexpression might be helpful in designing novel therapeutic strategies to stabilize shear stress-induced atherosclerosis.

## Figures and Tables

**Table 1:** Body weight, serum lipids, and hydroxyproline in three groups of mice.

Parameters	Mock	Lenti-EGFP	Lenti-P4H*α*1
	(*n* = 19)	(*n* = 20)	(*n* = 18)
Body Weight (g)	30.45 ± 0.39	31.12 ± 0.28	29.98 ± 0.76
TC (mmol/L)	1.24 ± 0.19	1.32 ± 0.21	1.28 ± 0.31
TG (mmol/L)	1.03 ± 0.09	1.12 ± 0.12	1.21 ± 0.14
LDL-C (mmol/L)	6.36 ± 0.71	6.54 ± 0.58	6.28 ± 0.56
HDL-C (mmol/L)	1.91 ± 0.35	1.89 ± 0.42	1.92 ± 0.31
Hydroxyproline (mg/mL)	1.78 ± 0.35	1.83 ± 0.41	3.09 ± 0.41^ab^

TC, total cholesterol; TG, triglycerides; LDL-C, low-density lipoprotein cholesterol; HDL-C, high-density lipoprotein cholesterol.

^a^ *p* < 0.05 versus mock group, ^b^ *p* < 0.05 versus lenti-EGFP group.
